# Melanotic Neuroectodermal Tumor of Infancy: A Rare Case Report

**DOI:** 10.7759/cureus.6521

**Published:** 2019-12-31

**Authors:** Atul Tiwari, Mohan L Yadav

**Affiliations:** 1 Department of Pathology, Mahatma Gandhi Medical College & Hospital, Jaipur, IND

**Keywords:** melanotic neuroectodermal tumor of infancy, vanillylmandelic acid, melanotic ameloblastoma, congenital melanocarcinoma, congenital pigmented epulis, benign tumor of infancy

## Abstract

Melanotic neuroectodermal tumor of infancy (MNTI) is a rare pigmented neoplasm of neural crest origin that most commonly arises in the maxilla of neonates and infants during the first year of life. We report the case of a three-month-old female child who presented with a 15-day history of intraoral swelling. Radiologically, the tumor was detected in the right maxilla, which was removed by wide local excision. Histopathological examination revealed the biphasic population of cells with melanin pigment deposition. Immunohistochemistry study was done confirming the diagnosis of MNTI.

## Introduction

Melanotic neuroectodermal tumor of infancy (MNTI) is a rare pigmented neoplasm of neural crest origin that most commonly presents in infants during the first year of life. It is predominant in males and most commonly arise in the maxillary region [1,2]. In the past, it has been known by a variety of names, such as congenital melanocarcinoma, melanotic epithelial odontoma, melanotic ameloblastoma, retinal anlage tumor, melanotic progonoma, pigmented adamantinoma, congenital pigmented epulis, and melanocytoma. Like other tumors of neuroectodermal origin, MNTI is frequently associated with elevated urinary excretion of vanillylmandelic acid (VMA), a metabolite of epinephrine and norepinephrine. Although an increase in VMA is helpful, this symptom alone is not diagnostic of MNTI [3,4].

The treatment of choice for this tumor is surgical excision. Because of its relatively high recurrence rate, cases of MNTI should be monitored closely during the postresection period and beyond. Although locally invasive, the risk of tumor metastasis is approximately 5% [3-5].

## Case presentation

A three-month-old female child presented with feeding difficulty and intraoral swelling noticed by her parents at two months of age. The swelling was progressively increasing in size. No history of fever, cold, and cough. The child was immunized adequately for the age with no delay in developmental milestones. General examination was unremarkable except for the presence of mild pallor.

On local examination, the swelling was well defined measuring 3 cm x 2 cm, firm in consistency with the smooth overlying surface. The swelling was non-tender and non-pulsatile. The swelling did not bleed on touch. No abnormality was found on systemic examination. A plain radiograph of the skull revealed a lytic lesion in the maxilla. On CT scan, an expanded soft tissue swelling in the right maxillary antrum was noticed. The expansion of the involved bone was seen. The chest radiograph was normal. In view of the above findings, a 24-hour urine sample was sent for estimation of VMA, which was found to be normal. A wide local excision of the tumor was done and sent for histopathological examination.

Grossly, the specimen received measures 3 cm x 3 cm x 2 cm and on the serial section, the cut surface showed a tumor measuring 2 cm x 2 cm x 1.5 cm. The cut surface of the tumor showed heterogeneous gray-black appearance.

**Figure 1:**
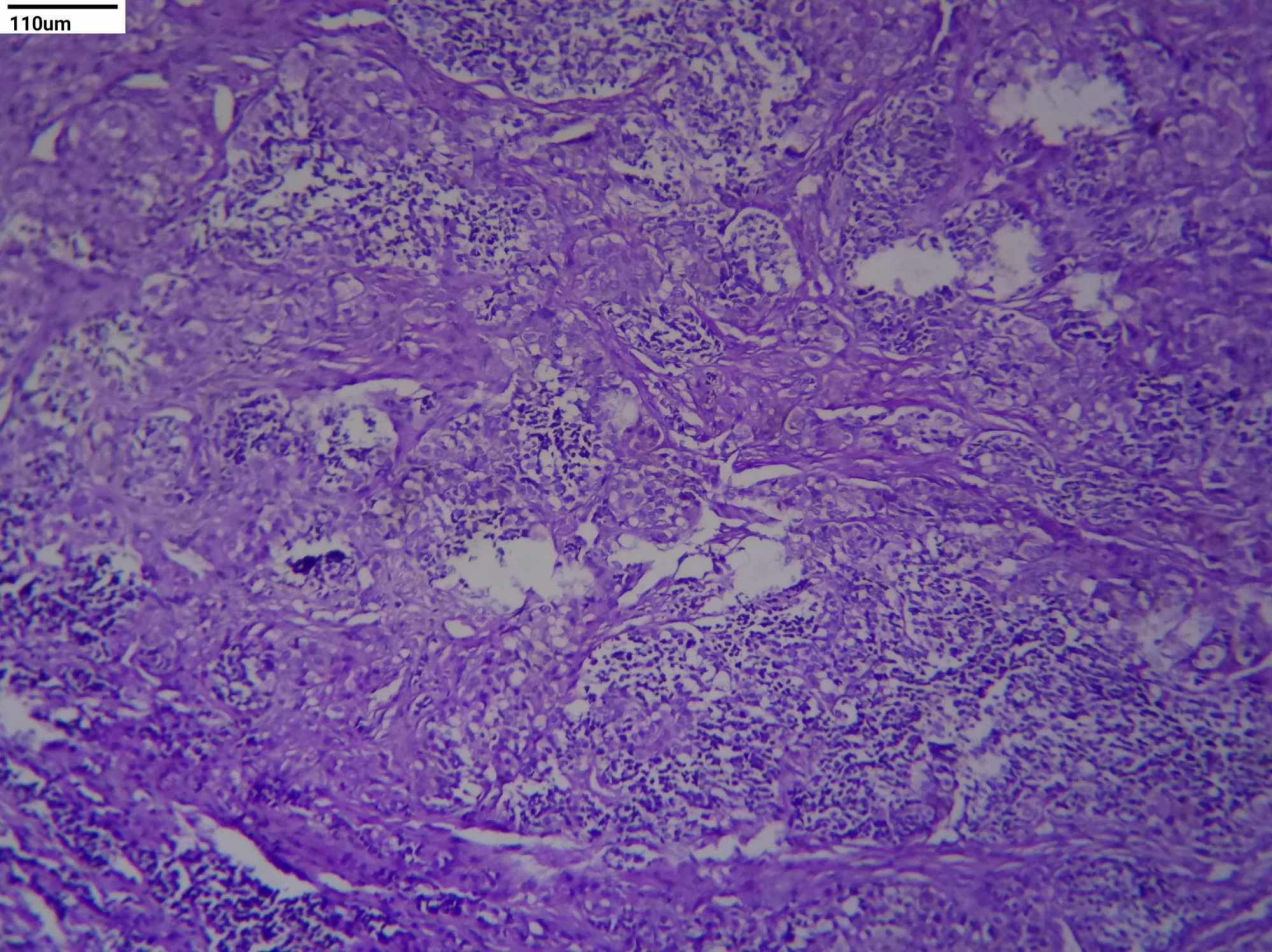
Tumor cells arranged in alveolar pattern separated by fibrovascular stroma (H&E, 10x) H&E: hematoxylin and eosin

**Figure 2:**
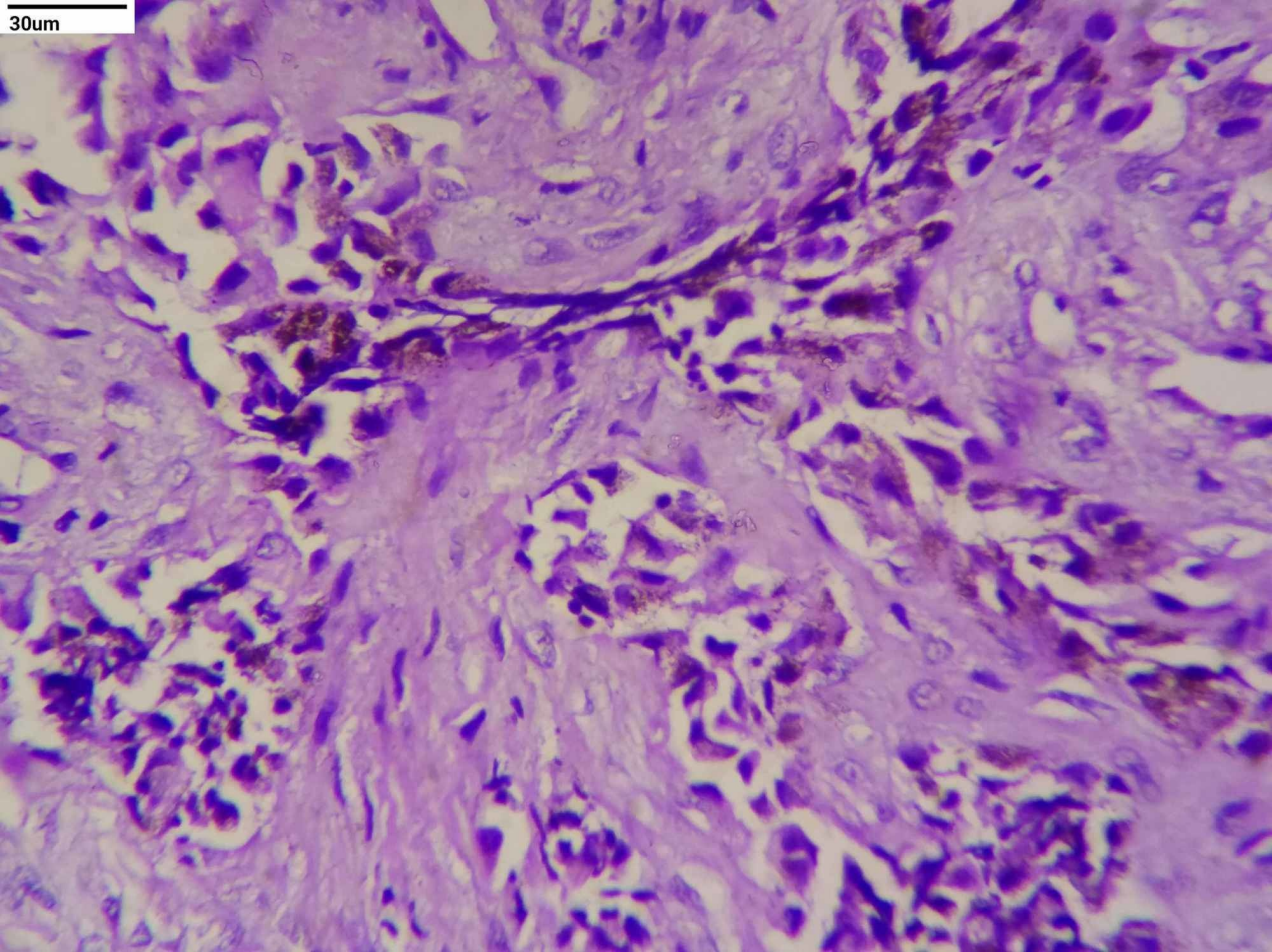
Tumor showing two distinct types of cells - large cells with abundant cytoplasm, round vesicular nucleus, and brown pigment; and small cells with scanty cytoplasm and hyperchromatic round nuclei (H&E, 40x) H&E: hematoxylin and eosin

On microscopic examination, the tumor was composed of cells arranged in alveolar pattern separated by a fibrovascular stroma. Two distinct types of cells were seen - large cells arranged peripherally with abundant cytoplasm, round vesicular nucleus, and brown pigment; and small cells with scanty cytoplasm and hyperchromatic round nuclei were seen in the center (Figures 1, 2). Histological features were consistent with a small round tumor, favoring the melanotic neuroectodermal tumor of infancy.

**Figure 3:**
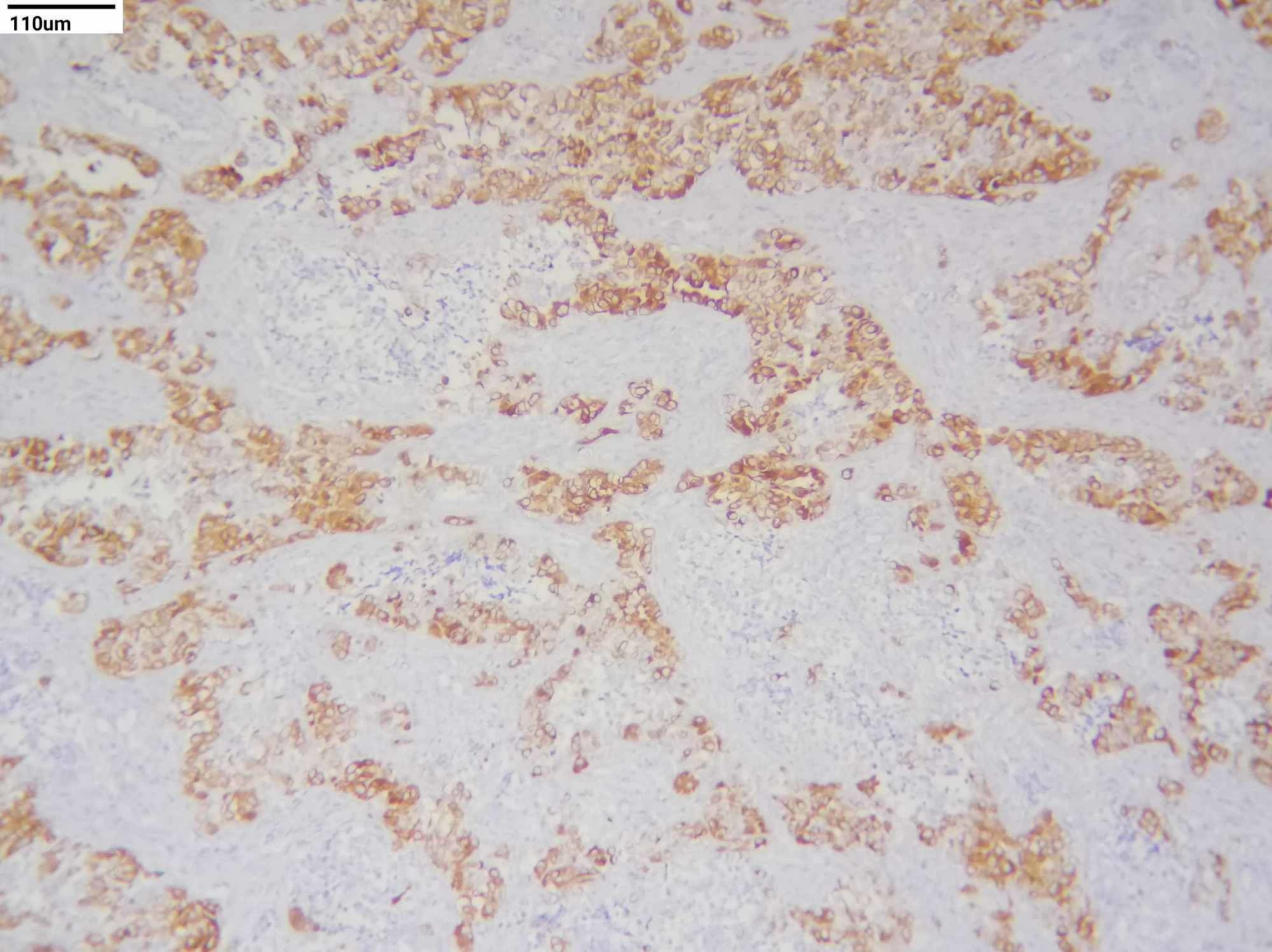
Immunohistochemistry staining - cytokeratin (10x)

**Figure 4:**
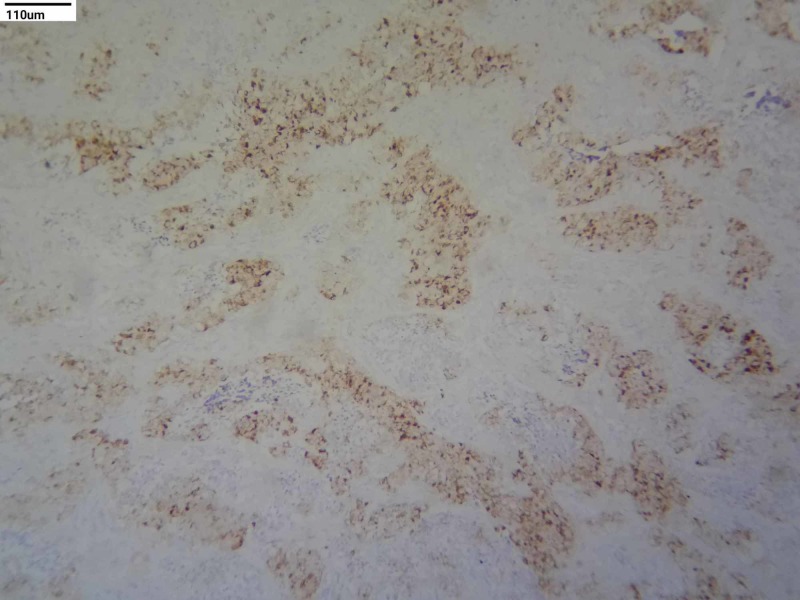
Immunohistochemistry staining - HMB-45 (10x)

**Figure 5:**
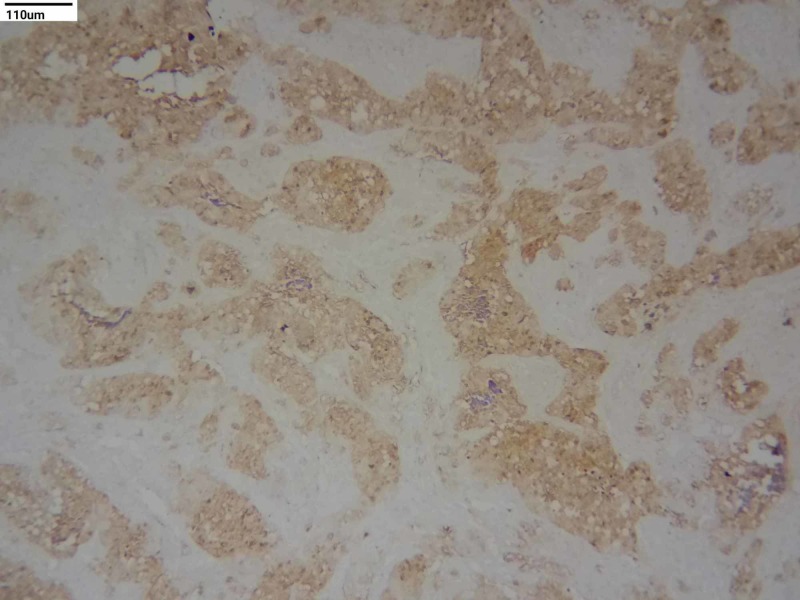
Immunohistochemistry staining - NSE (10x) NSE: neuron-specific enolase

**Figure 6:**
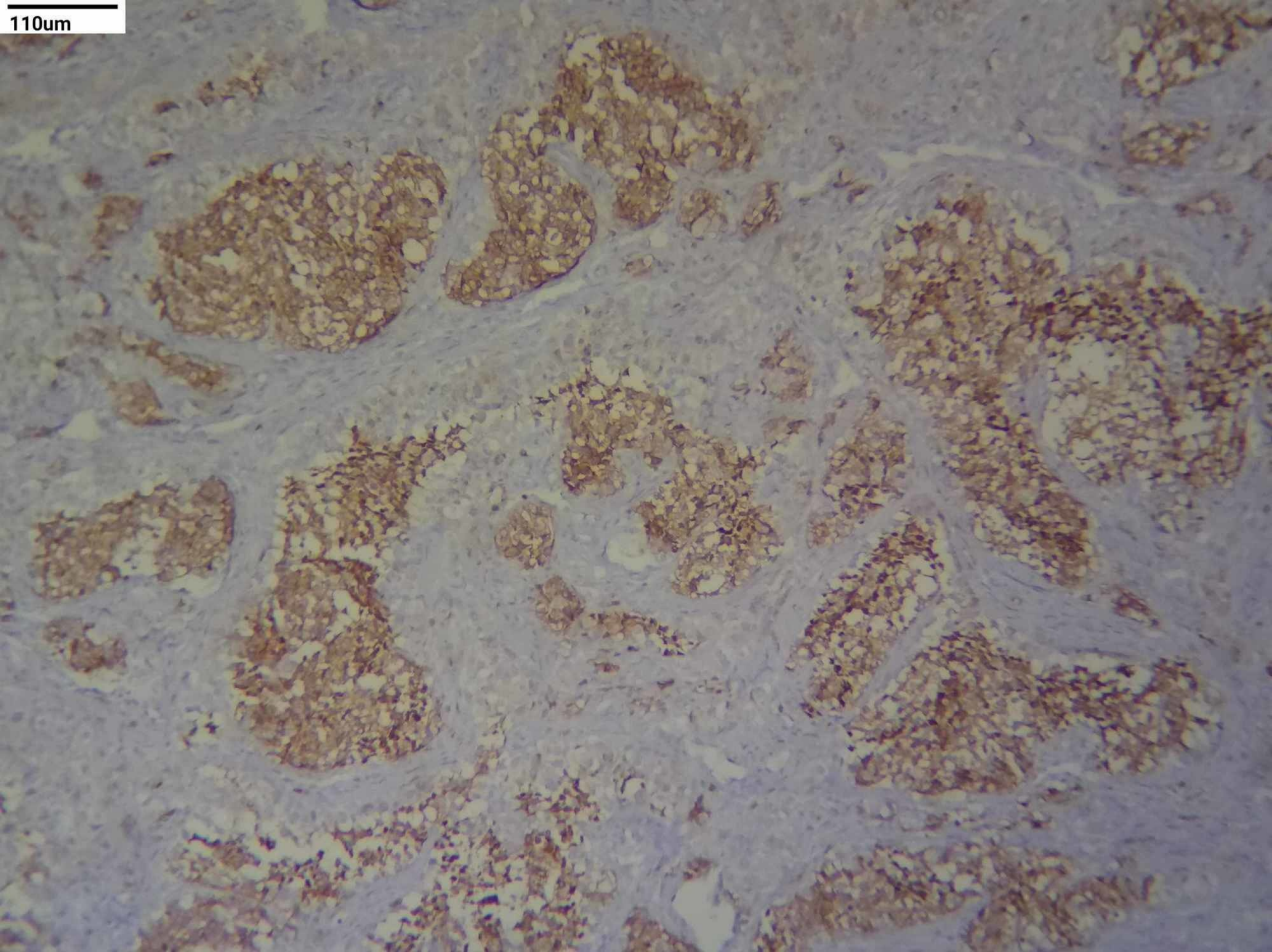
Immunohistochemistry staining - synaptophysin (10x)

Immunohistochemical (IHC) studies were done for the confirmation. Cytokeratin and HMB-45 were positive in large cells and negative for small cells. Neuron-specific enolase (NSE) and synaptophysin were positive in small round cells and negative in large cells (Figures 3-6). S100, CD99, and leukocyte common antigen (LCA) were negative in large and small cells.

Thus, a final diagnosis of MNTI was made by correlating HPE and IHC findings. This case was followed up for one year without any recurrence.

## Discussion

The first case of MNTI ever reported in the literature was designated as “congenital melanocarcinoma” by Krompecher in 1918 [6]. He described a pigmented tumor of the maxilla in a two-month-old infant. Since then it has been known by a variety of names, such as melanotic epithelial odontoma, melanotic ameloblastoma, retinal anlage tumor, melanotic progonoma, pigmented adamantinoma, congenital pigmented epulis, and melanocytoma. The term “melanotic neuroectodermal tumor of infancy” was recommended by Borello and Gorlin in 1966, when they reported a case of melanotic tumor in the maxilla of a three-month-old child who had increased urinary excretion of VMA before surgery, suggesting a neural crest origin [4].

MNTI typically occurs in infants <1 year and has a slight male predilection [7,8]. It most commonly occurs in the craniofacial region with a predominance for the maxilla [7]. Extramaxillary locations reported include the skull, mandible, long bones, epididymis, mediastinum, soft tissues of extremities, cheek, and brain.

MNTI was previously considered as the tumor of odontogenic origin, but IHC and ultrastructural studies and VMA production have confirmed its neural nature. Increased preoperative urinary VMA level is useful for diagnosing tumors of neural crest origin. The levels of VMA returned to normal following surgery and chemotherapy. The variable expression of IHC markers in different studies suggests that MNTI is a primitive neuroectodermal tumor with a polyphenotypic expression of neural and epithelial markers, and melanin production [5,9,10].

There is no typical biological behavior of MNTI. It clinically presents as a rapidly growing, painless, expansile, unencapsulated partly pigmented mass and is considered benign. Recent studies have indicated that the local recurrence rate following conservative resection is 10%-60%. It tends to occur as a single lesion. However, multiple lesions have also been reported [11]. Recurrence may occur due to the invasion of the tumor edge into the bone and difficulty in complete resection due to tumor with no envelope [8,10,12].

The differential diagnosis of MNTI includes nasopalatine cyst, globulomaxillary cyst, odontoma, ameloblastoma, odontogenic myxoma, rhabdomyosarcoma, Burkitt’s lymphoma, etc. However, this long list can be reduced to just a few based on the clinical and radiological features.

Conventional radiographs of bony lesions usually show radiolucency with or without irregular margins. CT scans typically reveal hyperdense masses, but hypodense variants have also been reported. CT scan accurately defines lesion extent and thus, provides a good basis for surgical planning. Magnetic resonance imaging shows a hypodense mass with focal hyperdense areas [8].

In addition to the typical clinical presentation, the cytology and histology are distinctive, showing a dual population of small neuroblastic-like round cells with condensed chromatin and larger melanin-containing polygonal epithelial cells [13]. Similar features are also noted in the present case.

IHC markers are useful in differentiating MNTI from various other small cell tumors of infancy such as embryonal rhabdomyosarcoma, in which desmin and myogenin are positive; Burkitt’s lymphoma where LCA is positive; and malignant melanoma which gives positivity to HMB-45 and S100. In the present case, IHC positivity for HMB-45 and NSE indicates melanocytic and neuroblastic differentiation of the tumor cells, respectively, thus confirming the diagnosis of MNTI [5,9].

The treatment of choice consists of complete surgical excision with negative margins. Chemotherapy is usually recommended only in cases of unresectable or metastatic disease.

## Conclusions

Although MNTI is usually a benign tumor, due to its rapid growth potential and locally destructive behavior, early detection and treatment can avoid further complications and this will support a favorable outcome for the patient. However, the rarity of the tumor often leads to a delay in diagnosis, resulting in less than the desired outcome. In the present case, early diagnosis and treatment prevented further complications and the patient was followed up for one year without any recurrence.
